# Simultaneous transmediastinal esophagectomy and thoracoscopic lobectomy for synchronous double cancers of the esophagus and lung: a case report

**DOI:** 10.1186/s44215-024-00150-w

**Published:** 2024-04-29

**Authors:** Kenji Kameyama, Koji Takao, Atsushi Shiozaki, Hitoshi Fujiwara, Tsunehiro Ii

**Affiliations:** 1 Department of Thoracic Surgery, Ayabe City Hospital, 20-1 Otuska, Aono-Cho, Ayabe-City, Kyoto, 623-0011 Japan; 2 Department of Surgery, Ayabe City Hospital, 20-1 Otuska, Aono-Cho, Ayabe-City, Kyoto, 623-0011 Japan; 3 Division of Digestive Surgery, Department of Surgery, Kyoto Prefectural University of Medicine, 465 Kajii-Cho, Kamigyo-Ku, Kyoto, 602-8566 Japan

**Keywords:** Synchronous double cancers of the esophagus and lung, Esophageal cancer, Lung cancer, Transmediastinal esophagectomy, Thoracoscopic lobectomy

## Abstract

**Background:**

Simultaneous surgery for synchronous double cancers of the esophagus and lung is so invasive that minimally invasive surgical procedures are preferred. For left lung cancer, there are few reports on simultaneous surgery due to the difficulty of performing radical esophagectomy only via the left thoracic approach and the high invasiveness of bilateral thoracotomy.

**Case presentation:**

A 65-year-old man who was diagnosed with synchronous double cancer of the esophagus and left lung underwent transmediastinal esophagectomy (TME) and thoracoscopic lobectomy (TSL) simultaneously. This procedure is advantageous because radical esophagectomy can be completed regardless of the side affected by the lung cancer, and respiratory function can be preserved by shortening the duration of differential lung ventilation and avoiding thoracotomy.

**Conclusion:**

This surgery could be a good treatment option for synchronous double cancers of the esophagus and lung in a highly proficient hospital.

**Supplementary Information:**

The online version contains supplementary material available at 10.1186/s44215-024-00150-w.

## Background

Since the incidence of synchronous double cancers of the esophagus and lung is low (1.7%) [1], the treatment strategy must be decided on a case-by-case basis. In the case of simultaneous surgery, minimally invasive surgical procedures are desirable, and the optimal approach for radical resection of both cancers must be selected. For left lung cancer, there are few reports on simultaneous surgery due to the difficulty of performing radical esophagectomy only via the left thoracic approach and the high invasiveness of bilateral thoracotomy [1, 2]. In this report, a new approach to simultaneous surgery using transmediastinal esophagectomy (TME) and thoracoscopic lobectomy (TSL) for a patient with synchronous double cancer is described.

## Case presentation

**Fig. 1:**
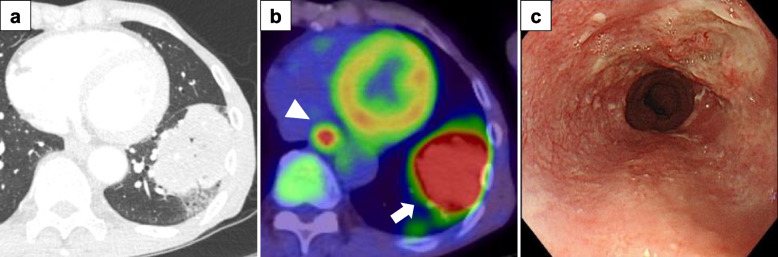
**a** CT image showing the solid tumor in the left lower lobe. **b** FDG-PET/CT image showing the accumulation of FDG in the lung tumor (arrow) and the lower thoracic esophagus (arrowhead). **c** Upper gastrointestinal endoscopy image showing a superficial, flat type of esophageal cancer

An asymptomatic 65-year-old man presented with an abnormal shadow on plain chest radiography. Computed tomography (CT) revealed a solid, 7.5-cm tumor in the left lower lobe, and CT-guided needle biopsy revealed a large cell carcinoma (Fig. 1a). F-18 fluorodeoxyglucose positron emission tomography (FDG-PET)/CT demonstrated the accumulation of FDG in the lung tumor (maximum standardized uptake value (SUVmax) 21.71) and in the lower thoracic esophagus (SUVmax 6.0) (Fig. 1b). Upper gastrointestinal endoscopy revealed a superficial, flat-type tumor involving the entire circumference of the lower thoracic esophagus, which was diagnosed as infiltrating into the submucosal layer on magnifying endoscopy and biopsy revealed a squamous cell carcinoma (Fig. 1c). There were no findings suggesting lymph node metastasis; therefore, the lung tumor was diagnosed as large cell carcinoma, staged as cStage IIIA (T4N0M0) according to the Union for International Cancer Control (UICC) TNM Classification, 8th edition. The tumor proportion score for lung cancer was less than 1. On the other hand, the esophageal tumor was diagnosed as squamous cell carcinoma, staged as cStage I(T1bN0M0). The combined positive score of the esophageal cancer exceeded 10. Given the lack of evidence-based chemotherapy for large cell carcinoma, and the absence of invasion into surrounding organs or lymph node metastasis, surgery was deemed the appropriate course of action, without the need for induction chemotherapy. Although definitive chemoradiation therapy was also considered as a treatment option for esophageal cancer, the patient expressed a preference for simultaneous surgical resection.

Although pulmonary function tests revealed restrictive ventilatory impairment (vital capacity (VC) 75.4%) and diffusion impairment (diffusing capacity of the lung for carbon monoxide (DLCO) 33.3%), The patient demonstrated a favorable performance status, as evidenced by their ability to easily climb stairs and cardiac function and other vital organs were also in good condition. Based on these assessments, we evaluated the patient to be at moderate risk for lobectomy and was deemed to have sufficient surgical tolerance. In addition, considering the adhesions and anatomical structure changes due to sequential surgery, simultaneous surgery for both cancers was planned. TME was performed first, followed by TSL (Supplemental video S1).

**Fig. 2:**
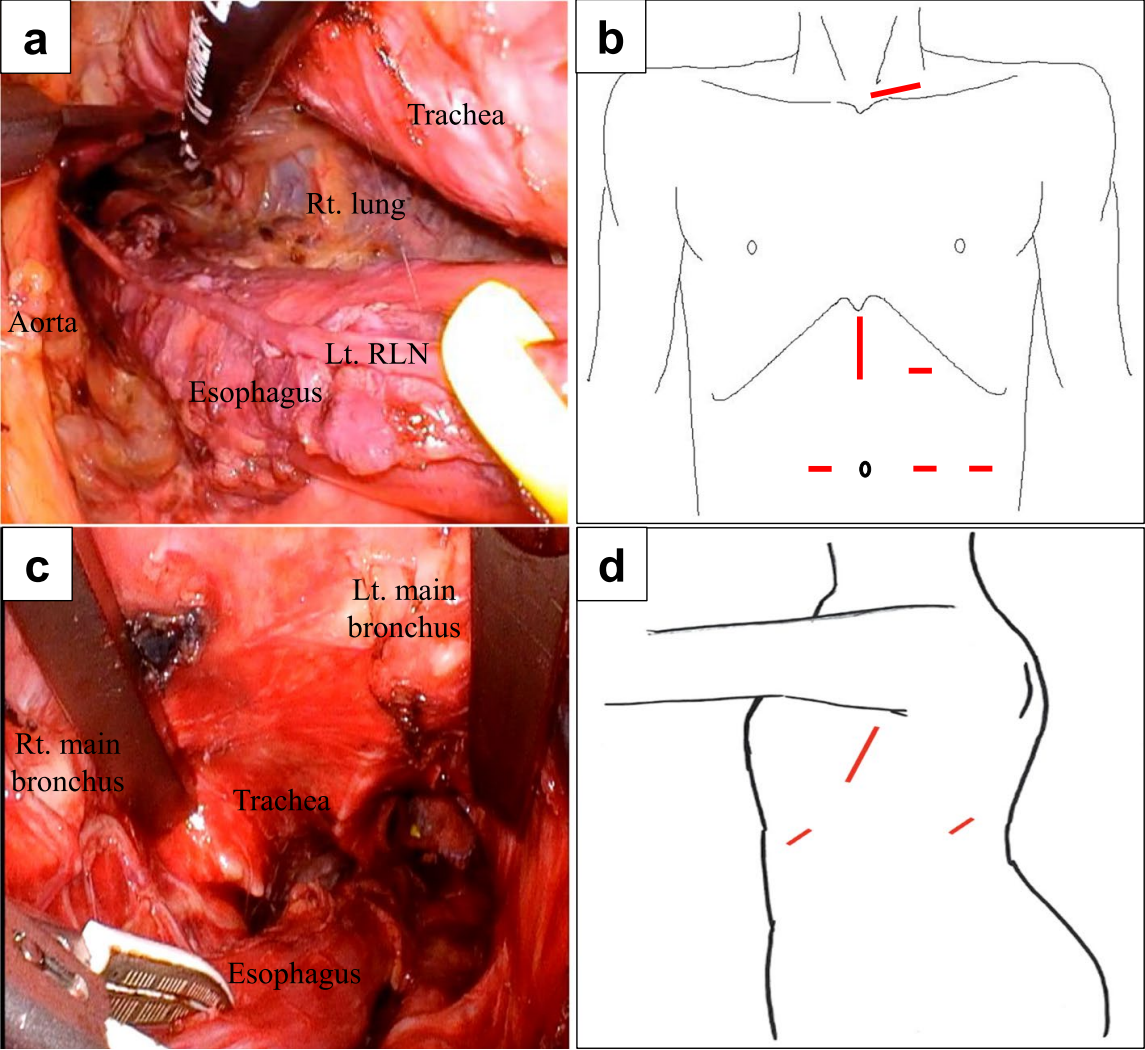
**a** View of the mediastinum from the left cervical approach after circumferential dissection of the esophagus. **b** Surgical incisions in the abdomen. **c** View of the mediastinum from the transhiatal approach. **d** Surgical incisions in the chest

For esophagectomy, the patient was placed in a supine position under general and epidural anesthesia. To avoid recurrent laryngeal nerve (RLN) injury during the transcervical approach, a single-lumen endotracheal tube with continuous intraoperative nerve monitoring with an NIM Response 3.0 (Medtronic, Dublin, Ireland) was used. First, a 5-cm skin incision was made on the left side of the neck, and the cervical esophagus was encircled. Following the dissection of the paraesophageal lymph nodes (LNs), a single-incision access platform (Hakko, Tokyo, Japan) with three 5-mm trocars was inserted into the cervical wound. Then, mediastinoscopic dissection was started with a forced pneumomediastinum of 10 mmHg. The esophagus was dissected circumferentially all the way down to the level of the inferior pulmonary vein, and the upper mediastinal LNs, including those along the left RLN and tracheal bifurcation (subcarinal and bilateral main bronchial LNs), were dissected (Fig. 2a). Next, abdominal and transhiatal mediastinal dissections were performed using a hand-assisted laparoscopic surgery technique. A 7-cm skin incision was made at the epigastrium; three 12-mm ports were inserted at the right peri-umbilicus, left subcostal abdomen, and left lateral abdomen; and a scope port was placed at the left peri-umbilicus (Fig. 2b). Following the dissection of the thoracic esophagus and mediastinal LNs from the neck and abdomen, gastric tube reconstruction was performed via the retrosternal route (Fig. 2c). After esophageal surgery, the intubation tube was replaced with a double-lumen tube, not using a bronchial blocker in order to allow for suctioning from within the bronchus in the event of bleeding from the periphery of the lungs during the lobectomy. The patient was repositioned to the right lateral decubitus position, and lobectomy was performed under differential lung ventilation. A 6-cm incision was made at the mid-axillary line of the 5th intercostal space (ICS), 1.5 cm ports were inserted at the posterior axillary line of the 8th ICS and the anterior axillary line of the 6th ICS, and a thoracoscopic left lower lobectomy was performed (Fig. 2d). The operative times for esophageal and lung surgery were 230 and 215 min, respectively. The total blood loss was 50 g. Following the surgery, the patient was transferred to the ICU and placed on a ventilator. At that time, the PaO2/FiO2 ratio exceeded 500. The following morning, the PaO2/FiO2 ratio remained above 500, so the patient was extubated on postoperative day 1 and evaluated for RLN palsy, but no palsy was noted. By the evening of the same day, supplemental oxygen was no longer necessary. No desaturation of percutaneous oxygen was observed during exertion. On postoperative day 20, the patient had aspiration pneumonia equivalent to Grade 2 Clavien-Dindo [3] and was treated with antimicrobial agents for one week. Since there was a period of fasting, the patient was discharged from the hospital on postoperative day 37.

The pathological diagnosis of esophageal cancer was squamous cell carcinoma with metastasis to the middle thoracic paraesophageal and paracardiac LNs, staged as pStage II (T1bN2M0) according to the UICC TNM classification, 8th edition. The pathological diagnosis of lung cancer was large cell carcinoma without LN metastasis, staged as pStage IIIA (T4N0M0). From postoperative day 47, two courses of CDDP + 5-FU were administered as adjuvant chemotherapy for esophageal cancer. After 10 months, multiple recurrences of lung cancer were detected in the liver and brain. We administered the immune-checkpoint inhibitor, ipilimumab, and nivolumab, but, unfortunately, he died of cancer only 1 month later.

## Discussion

According to previous reports, in cases of simultaneous surgery for double cancers of the esophagus and lung, esophageal surgery generally involves surgery through the thoracic cavity on the same side as the lung cancer side. In the case of left lung cancer, the aortic arch usually interferes with the completion of radical esophagectomy with LN dissection in the upper mediastinum [1, 2]. Therefore, the simultaneous combination of left thoracotomy for lung cancer and the conventional right thoracic approach for esophageal cancer or separate but sequential surgeries for esophageal and lung cancers must be chosen. In the present approach, radical esophagectomy can be performed regardless of the side affected by the lung cancer because it does not rely on a thoracic approach. As previously reported, TME offers a pneumomediastinum that facilitates radical esophagectomy and lymph node dissection comparable to open thoracotomy [4]. If TSL is performed first, it can be difficult to obtain a wide and favorable surgical field around the esophagus using pneumomediastinum. Moreover, pneumomediastinum results in an artificial pneumothorax, potentially causing subcutaneous emphysema and instability in respiratory and circulatory dynamics. Therefore, TME precedes TSL.

A major benefit of this surgery is its lesser invasiveness for respiratory function because of the avoidance of open and bilateral thoracotomy and the shorter duration of one-lung ventilation compared with conventional thoracic approach surgery [4]. Open or bilateral thoracotomy results in depression of respiratory function, which is one of the major factors related to postoperative respiratory complications [5, 6]. Differential lung ventilation also increases respiratory complications, so patients should be kept as short as possible [7]. In this case, despite preoperative compromised respiratory function, the patient exhibited an overall favorable postoperative respiratory status.

In conclusion, simultaneous TME and TSL resection could be good treatment options for patients with synchronous double cancers of the esophagus and lung at a highly proficient hospital.

## Supplementary Information

**Additional file 1: Supplemental video S1.** Simultaneous surgery of TME+TSL.

## Data Availability

All the data generated or analyzed during this study are included in this published article.

## Abbreviations

TME: Transmediastinal esophagectomy
TSL: Thoracoscopic lobectomy
CT: Computed tomography
FDG-PET/CT: F-18 fluorodeoxyglucose positron emission tomography/computed tomography
SUVmax: Maximum standardized uptake value
UICC: Union for International Cancer Control
VC: Vital capacity
DLCO: Diffusing capacity of the lung for carbon monoxide
RLN: Recurrent laryngeal nerve
LNs: Lymph nodes
ICS: Intercostal space
